# Timely diagnosis of atypical Japanese spotted fever: A case report

**DOI:** 10.1097/MD.0000000000044345

**Published:** 2025-09-05

**Authors:** Jiarong Li, Junning He, Yijie Yin, Yongfang Liu

**Affiliations:** a Department of Infectious Diseases, Third People’s Hospital of Chengdu, Chengdu, China; b Department of Clinical Medicine, North Sichuan Medical College, Nanchong, China.

**Keywords:** infection, Japanese spotted fever, metagenomic next-generation sequencing, *Rickettsia japonica*, spotted fever group rickettsiosis

## Abstract

**Rationale:**

Japanese spotted fever (JSF) is a rare tick-borne disease caused by *Rickettsia japonica*. Atypical manifestations and a lack of standardized diagnostic assays often result in delayed diagnosis and treatment, potentially leading to life-threatening complications.

**Patient concerns:**

A 57-year-old immunocompetent female from a region with no previously reported JSF cases presented with acute-onset high-grade fever (39.5°C), a generalized maculopapular rash, and systemic symptoms after participating in agricultural activities. The patient denied having any history of tick exposure and presented without eschar, leading to the initial misdiagnosis of respiratory infection.

**Diagnoses:**

Metagenomic sequencing (MetaCAP) technology enabled a definitive diagnosis by identifying *Rickettsia japonica*-specific DNA sequences in the patient’s blood. The genomic results completely aligned with the clinical presentation.

**Interventions:**

The patient was treated with doxycycline, which achieved rapid clinical resolution.

**Outcomes:**

The patient achieved full recovery with only residual lower-limb hyperpigmentation at the month follow-up, without disease recurrence.

**Lessons:**

This case demonstrates the diagnostic value of metagenomic testing for fevers of unknown origin. JSF should be a key consideration for agricultural and forestry workers presenting with compatible symptoms, even in nonendemic areas without documented insect bites. The optimal diagnostic approach combines clinical evaluation with advanced molecular testing to ensure the accurate identification and proper management of tropical febrile illnesses.

## 1. Introduction

*Rickettsia japonica* belongs to the spotted fever species group of the Rickettsiaceae family. Other diseases caused by different species of *Rickettsia*, such as scrub typhus (ST)^[1]^ and Q fever,^[2]^ are more prevalent in China, whereas Japanese spotted fever (JSF) is relatively rare. JSF was first discovered and reported in Japan in 1984.^[3]^ Since then, related reports have emerged from the Philippines,^[4]^ South Korea,^[5]^ and Thailand.^[6]^ The first case of JSF in China was identified in 2013.^[7]^ In the last decade, sporadic cases of JSF have been reported in various regions of China, including Hubei,^[8,9]^ Hunan,^[10]^ Shaanxi,^[11]^ Henan,^[12]^ and Zhejiang.^[13]^ JSF is transmitted to humans mainly through ticks,^[14]^ and disease onset is more common in summer and autumn, with mountainous and hilly regions being the main affected areas. Previously, JSF cases were considered rare and mostly occurred in remote mountainous and rural areas, leading to a lack of awareness among clinicians, which often resulted in misdiagnoses and missed diagnoses. Delayed treatment can lead to multiple organ failure, disseminated intravascular coagulation, and even death.^[15,16]^ A review of previous case reports revealed that the severity and potential lethality of JSF may have been seriously underestimated.

## 2. Case report

### 2.1. Patient information

The patient was a 57-year-old farmer from Wenchuan County, Sichuan Province, China, who was previously in good health, had no history of chronic diseases, and denied any history of insect bites or contact with animals. On the morning of June 15, 2024, the patient worked in fields on the mountain. She developed generalized muscle pain and fever 10 hours later, with the highest body temperature reaching 39.5°C. The following day, she developed small red papular rashes without itching her chest or back, which rapidly spread to her limbs. She also experienced sore throat, poor appetite, fatigue, and edema in both lower extremities. The patient received empirical cephalosporin treatment for 3 days, but her symptoms did not improve. The examinations conducted at the primary hospital revealed decreased lymphocyte (0.37 × 10^9^/L) and platelet (73 × 10^9^/L) counts and the disappearance of eosinophils (0 × 10^9^/L); a mild increase in C-reactive protein (CRP, 89 mg/L) and procalcitonin (0.86 ng/mL) levels; abnormal liver function, including significantly increased alanine aminotransferase (291 U/L), aspartate aminotransferase (309 U/L), and lactate dehydrogenase (285 U/L) levels; and abnormal coagulation function, indicated by increased D-dimer levels (8.6 mg/L), positive urine protein (3+), and moderate hyponatremia (127.6 mmol/L). Computed tomography revealed a small amount of bilateral pleural and pelvic effusion. Respiratory pathogen nucleic acid and blood culture test results were negative. The primary hospital considered the cause of the infection to be unknown and continued treatment with ticarcillin-clavulanate potassium. However, the patient’s condition worsened, with more severe fatigue and a poor appetite, and her indicators deteriorated. The patient was admitted to our hospital on June 25, 2024.

### 2.2. Clinical findings

**Figure 1.**
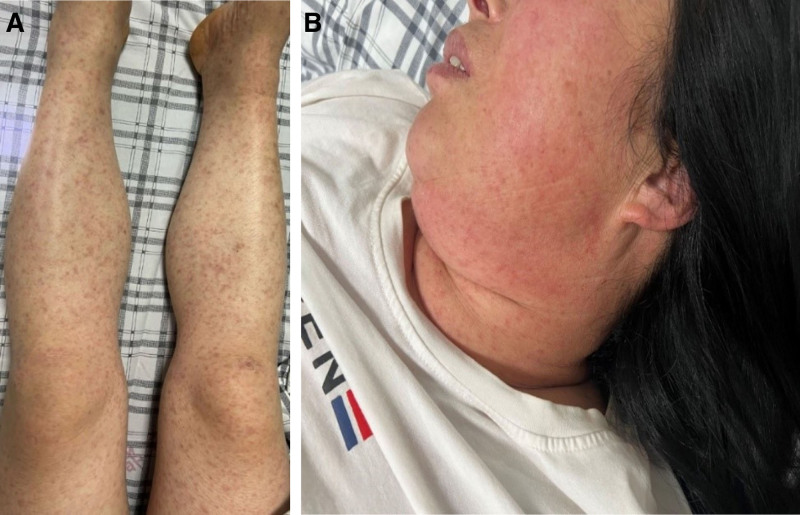
(A) Red rash on the trunk on the tenth day after onset. (B) Red rash on the face on the 10th day after onset.

The physical examination results were as follows: body temperature, 38.1°C; heart rate, 89 beats/minute; respiration rate, 20 breaths/minute; and blood pressure, 96/50 mm Hg. The patient had a clear mind; decreased mood; slurred speech; high bilateral eyelid edema; eyelid conjunctival congestion; facial flushing; diffuse distribution of red rice grain-sized maculopapules on the face, neck, chest, back, and limbs (including the palms and feet); prominent skin with partial pressure fading and blurred edges; and no insect-bite eschar (Fig. 1A and B). There was no superficial lymph node enlargement, and cardiopulmonary abdominal examination yielded no relevant results. Pitted edema of the extremities was also observed.

### 2.3. Diagnostic assessment and diagnosis

On the first day after admission, the patient underwent metagenomic sequencing (MetaCAP) of the pathogens identified in her blood. DNA fragments of *Rickettsia japonica* (sequence count: 227), Epstein–Barr virus (EBV) (sequence count: 2942), and cytomegalovirus (CMV) (sequence count: 9) were detected. Blood cultures were negative. Other supplementary auxiliary tests revealed negative results for the Weil-Felix test and significantly increased ferritin (>2000 ng/mL) and lactic acid (3.46 mmol/L) levels. Color Doppler ultrasound revealed enlarged lymph nodes in the left supraclavicular fossa, bilateral axillary fossae, and bilateral inguinal regions with slightly abnormal structures. The patient was ultimately diagnosed with JSF.

### 2.4. Treatment and follow-up

The patient was treated with 0.1 g of oral doxycycline every 12 hours for 9 days, along with liver protection therapy. The patient’s fever subsided on the second day of doxycycline treatment. Seven days later, the red rash partially faded and turned brown, and the symptoms of generalized muscle pain, sore throat, poor appetite, and fatigue gradually improved. Reexamination revealed improvements in the indicators (Table 1). After discharge, the patient continued taking oral liver-protective drugs. One month later, her liver function returned to normal. At the 6-month follow-up visit, the patient still had a small amount of brown pigmentation on the skin of both lower extremities but no other symptoms of discomfort.

**Table 1:** Patient test results on the 5th, 10th, and 17th days after onset.

Result	Normal range	D5	D10
WBC count (×10^9^/L)	3.5–9.5	5.62	7.3
N%	40–75	85	80
E%	0.4–8.0	0	0
L%	20–50	6.5	12.6
PLT count (×10^9^/L)	125–350	73	60
ALT (U/L)	<40	291	234
AST (U/L)	<40	309	126
LDH (U/L)	109–245	285	346
D-dimer (mg/L)	< 0.5	8.6	11
CRP (mg/L)	< 0.5	89	22
IL-6 (pg/mL)	< 7		34
PCT (ng/mL)	<0.25	0.86	0.6
Proteinuria	Negative	3+	Negative
Ferritin (ng/mL)	15–200		>2000
Lactic acid (mmol/L)	0.5–1.7		3.46

ALT = alanine aminotransferase, AST = aspartate aminotransferase, CRP = C-reactive protein, E% = eosinophil ratio, IL-6 = interleukin-6, L% = lymphocyte ratio, LDH = lactate dehydrogenase, N% = neutrophil ratio, PCT = procalcitonin, PLT = platelet, WBC = white blood cell.

## 3. Discussion and review of the literature

Typical symptoms of JSF include high fever, rash, and eschar, with a body temperature often >38.5°C. The rash appears as diffuse erythema of the whole body without pain or itching; the size of the spots is approximately that of the rice grains or soybeans, and the edges of the spots are blurred. The rash usually spreads rapidly from the torso to all parts of the body within a few hours. The rash disappears after remission or becomes pigmented.^[17]^ In a Japanese study, 57.1% (40/70) of 70 patients had all 3 signs (the triad), 32.9% of patients had 2 signs, 5.7% of patients had eschar and fever, and 27.1% had rash and fever.^[18]^ A patient with severe JSF in Guangyuan city,^[19]^ Sichuan Province, received delayed treatment due to the absence of eschar, which suggests that the presence of 2 of the triad symptoms should be considered to indicate JSF disease, even without evidence of insect bites.

Common nonspecific clinical manifestations of JSF include headache, rickets, fatigue, muscle pain and loss of appetite, and complications include pneumonia, noncardiogenic pulmonary edema, meningoencephalitis, central nervous system diseases, disseminated intravascular coagulation, severe shock, multiple organ failure, and even death.^[8,20–22]^ In addition to the symptoms mentioned in previous reports, the patient in this case also presented with limb edema, eyelid edema, conjunctival congestion, superficial lymph node enlargement, pleural and pelvic effusion, no headache, and no insect-bite eschar. Severe edema and conjunctival congestion were uncommon in previous reports.

JSF is often difficult to distinguish from the tick-borne diseases, ST and severe fever with thrombocytopenia syndrome (SFTS) because of their similar clinical manifestations. Compared with that of ST, the JSF eschar is smaller and more easily ignored and can disappear spontaneously within a few days. Erythema on the palms and soles of the feet is a unique symptom of JSF and is rare in ST patients. Local or systemic nodal lesions occur in almost all ST patients, whereas in JSF patients, this phenomenon is less pronounced. Hypotension is more common in JSF patients than in ST patients.^[23]^ Although these diseases are difficult to distinguish clinically, they respond well to tetracycline and quinolones. SFTS is caused by infection with the SFTSV RNA virus. Compared with JSF patients, SFTS patients have more severe manifestations, more complications, a higher mortality rate (31.7% vs 5%),^[24]^ more central nervous system involvement, more lung involvement, and more secondary bacterial and fungal infections. Two Korean studies proposed a scoring system to distinguish SFTS from ST. The variables used included altered mental status, leukopenia (white blood cell count <4000/µL), thrombocytopenia (platelet count <1 × 103/µL or 80 × 103/µL), a prolonged activated partial thromboplastin time (>35 seconds), and a normal CRP level (≤1.0 mg/dL). The lack of response to tetracycline and quinolones is another distinguishing characteristic. A retrospective Japanese study suggested that a normal CRP level alone could be used to clearly distinguish SFTS from JSF and that the absence of rash and leukopenia may aid in diagnosis.

The most relevant laboratory test results were an increased CRP level and a significantly decreased eosinophil count, which were detected in almost all JSF patients. The positivity rate of JSF diagnostic screening, which is based on fever, rash, an increased CRP level, and eosinophilia, is 90.9%.^[17]^ The most common cytokine change is an increase in Interleukin- 6 (IL-6) and Interferon-γ (IFN-γ) levels in the serum of JSF patients; a high IFN-γ concentration inhibits the differentiation of eosinophils, which may explain the observed decrease in eosinophils.^[25]^ Other major laboratory findings included leukocytosis or leukopenia, thrombocytopenia, lymphocytopenia, elevated alanine aminotransferase and aspartate aminotransferase levels, an elevated D-dimer level, and positive urine protein. Most patients have an increased serum lactate dehydrogenase level and hypoproteinemia, followed by hyponatremia, hypokalemia, anemia, hyperbilirubinemia, and an increased serum creatine kinase level.^[12]^ The white blood cell count and fibrinogen degradation product, CRP, and soluble interleukin-2 receptor (sIL2-R) levels were significantly increased in severe cases, with sIL2-R levels of 10,000 U/mL or higher produced by activated lymphocytes. These findings suggest that sIL2-R levels >10,000 U/mL can be used as a marker of poor prognosis.^[26]^

The diagnosis of JSF is challenging, traditional methods have limitations, and emerging technologies offer new approaches. *Rickettsia japonica* is an intracellular bacterium with strict requirements for growth conditions and cannot be obtained by routine laboratory culture. Indirect immunofluorescence assay (IFA), enzyme-linked immunosorbent assay (ELISA), the Weil–Felix test, and the latex agglutination test are the most common laboratory diagnostic methods used for the serological detection of *Rickettsia*. Among these methods, indirect immunofluorescence is widely used at home and abroad because of its specificity. However, these methods have the disadvantages of species uncertainty and early diagnosis. There is currently no antibody detection method for *Rickettsia japonica* in China. Early diagnosis of JSF depends on the clinical symptoms and judgment of the effects of specific antibiotics, and the diagnosis is often missed. With the progress of modern medical technology, gene sequencing technologies, such as metagenomic next-generation sequencing (mNGS) and targeted NGS (tNGS), have been widely used for microbial identification in recent years and have shown significant sensitivity and specificity in the diagnosis of infectious diseases.^[27]^ However, mNGS may miss the detection of some pathogens and is expensive, and the range of tNGS pathogen profiles is limited. Given these limitations, we adopted an innovative pathogen detection technology called pathogenic MetaCAP. This technology combines probe capture technology with NGS, covers a broad spectrum, has high sensitivity, covers the whole genome of RNA viruses, is compatible with viral homologous mutations, covers drug resistance sites in pathogens, and is highly cost effective.^[28]^ MetaCAP has the ability to detect DNA and RNA simultaneously and has broad application potential in the identification of pathogens causing insect-borne diseases. For patients in whom clinical manifestations of *Rickettsia* infection and RNA virus infection cannot be ruled out, the early use of MetaCAP technology is an efficient and accurate strategy for diagnosis.

EBV and CMV viral nucleic acid fragments can be detected in blood using MetaCap technology, but this does not indicate viral infection. Since the population is generally susceptible to EBV and CMV, the virus may be latent in lymphocytes. *Rickettsia japonica* is a macrophagophilic bacterium, and infected macrophages in the acute phase are stimulated to activate Th1 cells, which may allow viral replication.^[25]^ The manifestations in this case were inconsistent with those of EBV and CMV infections, and the patient’s symptoms improved rapidly after doxycycline treatment. The virus is not believed to cause fever in patients.

In addition to blood, skin lesions resulting from a rash or eschar should be sent for epidemiological microbiological tests, which is more sensitive than blood tests, even after antibiotic use.

Doxycycline is the first-line treatment for JSF, and minocycline is effective. Tetracyclines combined with quinolones have shown good efficacy in several cases of severe infections. In addition, steroids can be used to inhibit the early inflammatory response; however, the specific dose and duration of treatment need to be further determined through case studies. The treatment should be adjusted according to the specific conditions of patients.

## 4. Conclusion

In summary, JSF is an insect-borne disease with a low incidence and is easily misdiagnosed or missed. The clinical manifestations of JSF are complex and diverse, and multiple types of organ damage are likely to occur. Atypical cases are also common. Clinicians often lack awareness of this disease; as such, we suggest the following algorithm for JSF diagnosis: When a patient has specific complaints (fever, rash, eschar, or the combination of any 2 complaints), it is important to collect a detailed epidemiological history (history of activity in hilly, forested, and mountainous areas during the epidemic season) and conduct a comprehensive physical examination (focusing on the appearance of rash and suspicious eschar traces). If JSF is suspected, doxycycline should be administered immediately. Moreover, routine blood tests, as well as liver, kidney, and coagulation function tests and CRP levels should be performed. It is not necessary to wait for the test results, but it is necessary to send blood or skin samples from a rash or eschar for polymerase chain reaction or pathogen MetaCAP before the use of antibiotics to confirm the etiology. If there are no initial positive signs, close dynamic observation is necessary. Rapid and accurate diagnosis can aid in early etiological treatment, thereby reducing the potential risk of death. This case report highlights the importance of early recognition and prompt treatment of JSF and provides a reference for the management of similar cases in the future.

## Author contributions

**Conceptualization:** Jiarong Li.

**Investigation:** Jiarong Li, Junning He.

**Methodology:** Jiarong Li, Yijie Yin.

**Visualization:** Jiarong Li, Yongfang Liu.

**Writing – original draft:** Jiarong Li, Junning He.

**Writing – review & editing:** Yongfang Liu.
